# Hybrid Fiber Optic Sensor, Based on the Fabry–Perot Interference, Assisted with Fluorescent Material for the Simultaneous Measurement of Temperature and Pressure

**DOI:** 10.3390/s19051097

**Published:** 2019-03-04

**Authors:** Xiaofeng Jiang, Chun Lin, Yuanqing Huang, Kan Luo, Jianhuan Zhang, Qingshan Jiang, Chentao Zhang

**Affiliations:** 1 School of Information Science and Engineering, Fujian University of Technology, Fuzhou 350118, China; 19861937@fjut.edu.cn (X.J.); luokan@fjut.edu.cn (K.L.); 2 School of Aerospace Engineering, Xiamen University, Xiamen 361005, China; linchun@xmu.edu.cn (C.L.); yqhuang@xmu.edu.cn (Y.H.); aeolus@xmu.edu.cn (J.Z.); 3 Research and Development Center for Industrial Automation, Technology of Fujian Province, Fuzhou 350118, China; 4 Fujian (Quanzhou)-HIT Research Institute of Engineering and Technology, Quanzhou 362000, China; qs-bctf@163.com

**Keywords:** hybrid optic fiber sensor, Fabry–Perot interference, fluorescent lifetime

## Abstract

Herein we design a fiber sensor able to simultaneously measure the temperature and the pressure under harsh conditions, such as strong electromagnetic interference and high pressure. It is built on the basis of the fiber-optic Fabry–Perot (F–P) interference and the temperature sensitive mechanism of fluorescent materials. Both halogen lamps and light-emitting diodes (LED) are employed as the excitation light source. The reflected light from the sensor contains the low coherent information of interference cavity and the fluorescent lifetime. This information is independent due to the separate optical path and the different demodulation device. It delivers the messages of pressure and temperature, respectively. It is demonstrated that the sensor achieved pressure measurement at the range of 120–400 KPa at room temperature with a sensitivity of 1.5 nm/KPa. Moreover, the linearity of pressure against the cavity length variation was over 99.9%. In the meantime, a temperature measurement in the range of 25–80 °C, with a sensitivity of 0.0048 ms/°C, was obtained. These experimental results evince that this kind of sensor has a simple configuration, low-cost, and easy fabrication. As such, it can be particularly applied to many fields.

## 1. Introduction

Fiber-optic sensors have attracted increased interest for pressure monitoring, in many fields, due to advantages including their light weight, small size, corrosion resistance, high sensitivity, large dynamic response range, and strong anti-electromagnetic interference capability [1,2]. Due to their small size and strong anti-interference ability, Fabry–Perot (F–P) sensors have become one of the most promising fiber-optic sensors [3,4,5]. Moreover, their applications have been found in a broad range of areas, including industry [6,7], aerospace [8], and national defense [9,10]. More importantly, they have a rather high resolution, which is the most prominent feature of the F–P sensors.

In recent years, micro-electro-mechanical systems (MEMS) have been widely used in the manufacture of fiber-optic F–P sensors in order to make the product compact and with good consistency. Meanwhile, batch production can be easier [11,12,13]. However, the common MEMS fiber-optic F–P sensors cannot achieve accurate pressure measurements in actual applications due to their high sensitivity to temperature [14,15]. Much effort has been put into compensating the influence of temperature during pressure measurement [16,17]. Some novel fiber-optic F–P sensors with hybrid configurations have been designed and manufactured, such as one sensor integrating two cascaded F–P cavities [18,19,20], two fiber Bragg gratings (FBG) with different sensitivity coefficients [21,22], and attaching FBG to an extrinsic F–P interferometer [23,24,25]. These approaches have improved the accuracy of pressure measurement, however they are very complex in configuration and difficult to manufacture.

In this paper, we present a low-cost and easily fabricated fiber-optic sensor for simultaneous measurement of both temperature and pressure. Fluorescent material is introduced into fiber-optic F–P sensor to obtain temperature information owing to its temperature-sensitive mechanism. This hybrid sensor can achieve precision measurement of temperature and pressure without complex structures, leading to not only a decrease in the manufacturing cost but also a cross-sensitivity falling between temperature and pressure. In this contribution, we investigated the characteristics and parameter demodulation methods of hybrid fiber-optic F–P sensors for simultaneous measurements of temperature and pressure. The marriage of fiber-optic F–P sensors and temperature-sensitive fluorescent materials, to the best of our knowledge, is a new field worth investigating.

## 2. Theory and Experiments

### 2.1. Theory of the Hybrid Fiber-Optic Sensor

**Figure 1:**
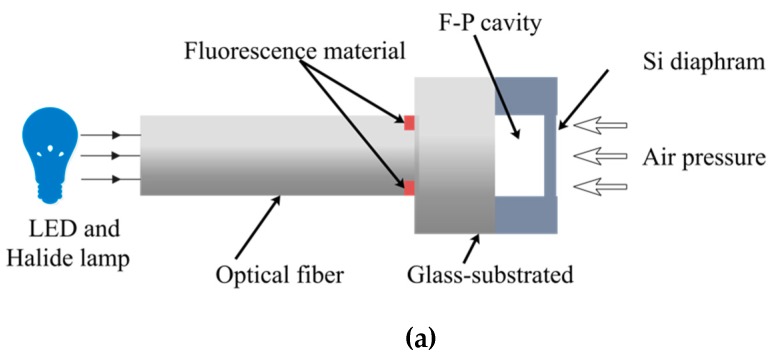

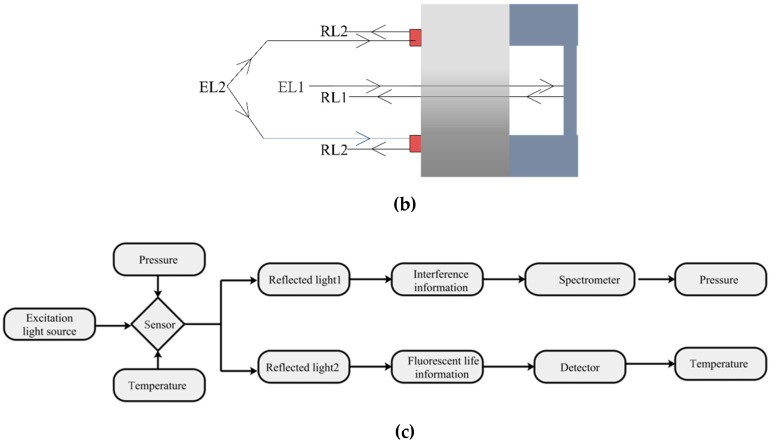
Schematic diagram of (**a**) configuration; (**b**) optical path; (**c**) working principle of the hybrid fiber-optic F–P sensor.

The hybrid fiber-optic sensor is designed based on the principle of F–P interference and the temperature sensitive mechanisms of fluorescent materials. Figure 1a–c depicts the schematic diagram of configuration, the optical path, and the working principle of the hybrid fiber-optic sensor, respectively. The excitation light source is comprised of a halide lamp and an LED with a central wavelength of 415 nm. The excitation light irradiates to the end of optical fiber and propagates into the hybrid fiber-optic sensor. The dual reflected lights are generated from F–P cavity (reflected light 1) and fluorescent material (reflected light 2), providing the information about the low coherence of the interference cavity and the lifetime of the fluorescent material. These two kinds of information are independent since they propagate along separate optical paths. They can be translated into the values of pressure and temperature by demodulation devices. Both single-mode and multi-mode optical fibers can be employed for light propagation in the sensor. Accordingly, it has good compatibility with the existing fiber-optic communication systems. Last but not least, two sensing probes for the measurement of pressure and temperature are integrated into one sensor, making it compact, low-cost, and easy to install.

### 2.2. Theory, Experimental Setup, and Method of Pressure Measurement

**Figure 2:**
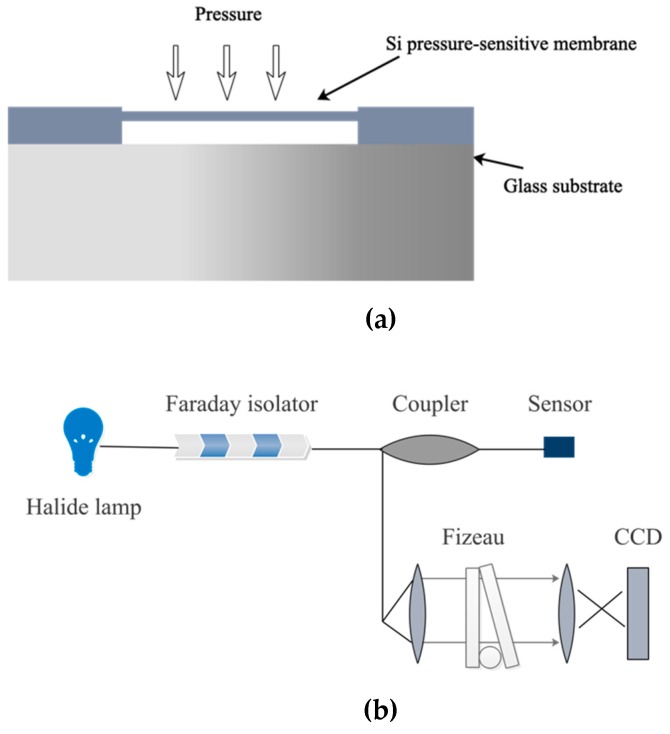
Schematic drawing of (**a**) the pressure-sensitive probe and (**b**) the experimental setup for pressure demodulation.

The pressure-sensitive probe of the hybrid fiber-optic F–P sensor is shown in Figure 2a. According to the principle of elasticity, the relationship of center deflection (*L*) of pressure-sensitive membrane and the pressure (*P*) applied on it is [26] as follows:

(1) $$\;L=\frac{3P\left(1-\mu^{2}\right)}{16Eh^{3}}{\left(R^{2}-r^{2}\right)}^{2}$$

where μ and *E* are the Poisson’s ratio and Young’s modulus of pressure-sensitive membrane, respectively; *h* and *R* are the thickness and radius of pressure-sensitive membrane; and *r* gives the radius of pressure-sensitive membrane [27,28].

The mechanical sensitivity (*S*) of pressure-sensitive membrane at the central point is calculated as follows:

(2) $$\;S=\frac{dL_{max}}{dP}=\frac{3\left(1-\mu^{2}\right)}{16Eh^{3}}R^{4}$$

The main factors that determine the variation of the pressure-sensitive membrane’s deflection are the radius and thickness of pressure-sensitive membrane. A larger radius or a smaller thickness will result in higher sensitivity.

The pressure-sensitive membrane is comprised of a silicon pressure-sensitive membrane and a glass substrate, which form a vacuum F–P cavity by anodic vacuum bonding. The incident light is coupled into the fiber and then propagates to the glass substrate. A part of light passes through the glass substrate and goes into the F–P cavity, where it propagates back and forth and leads to a multi-beam interference. The other part of light is reflected by the interface of the glass substrate and F–P cavity and then returns along the original propagation path. Finally, these two parts of light interfere. The interference signal carries the information along the length of the F–P cavity. The interference signal varies with the length of F–P cavity, which is determined by the pressure applied on the pressure-sensitive membrane. Therefore, the variation of pressure can be obtained by demodulating the interference signals.

Figure 2b shows the experimental setup and operating principle of pressure demodulation. A tungsten halogen lamp is employed as a broadband light source. Both sides of the Fizeau interferometer have 30% reflective coatings. The broadband light emitting from the tungsten halogen lamp passes through the 1 × 2 coupler and then enters the fiber-optic F–P sensor. The reflected light from the sensor is collimated by the lens and propagates into the Fizeau interferometer. The intensity distribution of output light from the Fizeau interferometer is recorded by a CCD sensor, which is essentially the same as the spatial distribution intensity of reflected light from the hybrid fiber-optic F–P sensor, after cross-correlation calculation. The Faraday isolator is used to prevent the reflected light from feeding back into the light source, which would introduce noise to subsequent demodulation. The interference signal is reconstructed via an inverse discrete wavelet transform to reduce the noise. The reconstructed interference signal is analyzed by a cross-correlation algorithm for the demodulation of cavity length [29,30,31]. 

### 2.3. Theory, Experimental Setup, and Method of Temperature Measurement

The working principle of temperature measurement of the hybrid fiber-optic F–P sensor is based on the fluorescence phenomenon. For some fluorescent material, the fluorescence intensity and fluorescence decay lifetime are related to the temperature and the intensity of excited light.

According to the principle of Boltzmann distribution law, the fluorescence lifetime *τ* is given by [32] as follows:

(3) $$\tau =\frac{1+\frac{g_{2}}{g_{1}}\exp\left(\frac{-\Delta E}{KT}\right)}{\frac{1}{\tau_{1}}+\frac{g_{2}}{\tau_{2}g_{1}}\exp\left(\frac{-\Delta E}{KT}\right)}$$

where *g*_1_ and *g*_2_ are the degeneracies level 1 and level 2, respectively, *K* is Boltzmann’s constant, *T* is the Kelvin temperature, *E* is static energy, and *τ*_1_ and *τ*_2_ are the first order fluorescence lifetime and second order fluorescence lifetime, respectively. Therefore, the variation of temperature can be achieved by demodulating the fluorescence lifetime, *τ*. 

**Figure 3:**
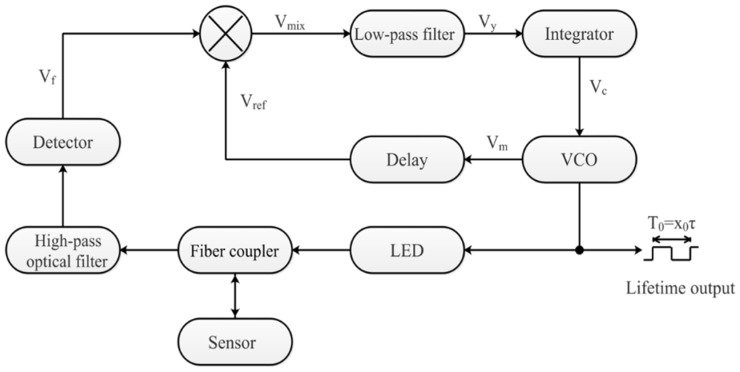
Sketch map of demodulation for fluorescence lifetime.

Figure 3 shows the working principle of demodulating fluorescence lifetime, *τ*, based on a phase-locked detection system with the modulation of a single reference pulse. A voltage-controlled oscillator (VCO) generates square wave signals, Vm, with a duty cycle of 50% and a repetition rate of 10. Vm serves as excitation signal to periodically modulate the excitation light from the LED. The excitation light passes through the fiber coupler and then enters the fiber-optic F–P sensor, which excites the sensor to generate fluorescence. The fluorescence propagates into the fiber coupler and enters the detector. The detector is a Si amplified photodetector (Thorlabs PDA 100). A high-pass filter in the front of detector is used to filter out of the excitation light. The fluorescence is converted to voltage, Vr, by the detector. Vm is delayed by a fixed period (αT) to form a reference signal, Vref. Vref is mixed with Vr to form a mixed signal, Vmix. Vmix is passed through a low-pass filter. The output of low-pass filter, designated as Vy, is given by the following:

Vy = f(x,α)(4)

where *x* is the ratio of the period of the modulation signal *T*_0_, given by

*x* = *T*_0_/*τ*. (5)

Vy is forwarded to an integrator and then the output of integrator Vc is fed back to control the VCO output until it tends to a stable frequency, which is the “locked” state for a phase-locked detection system. In the “locked” state, Vy = 0, *τ* can be calculated with Equations (5) and (6).

### 2.4. Manufacture of a Hybrid Fiber-Optic F–P Sensor

**Figure 4:**
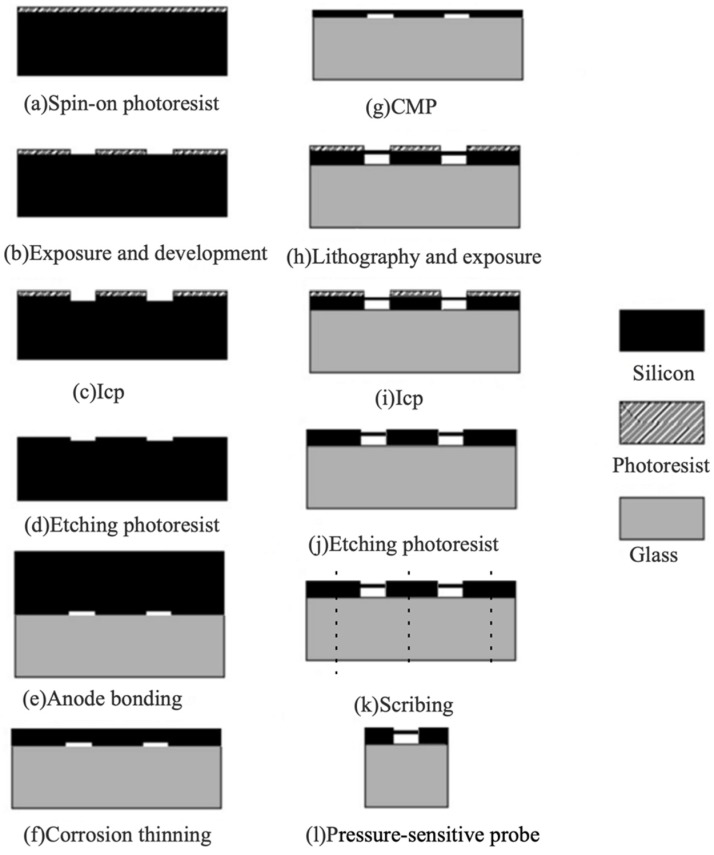
Manufacturing process of the pressure-sensitive probe.

The manufacturing process of a pressure-sensitive probe is shown in Figure 4. Firstly, a 4-inch silicon wafer is spin-coated with a photoresist AZ5214E (Figure 4a). Then lithography and developing processes are conducted to selectively remove the photoresist as the patterns of mask. Rectangle arrays are formed on the photoresist (Figure 4b). The remaining photoresist layer is utilized as the mask for inductive couple plasma (ICP) etching to transfer the pattern from the rectangle array to the surface of the silicon wafer (Figure 4c). After ICP etching, the remaining photoresist layer is removed by organic solvent (Figure 4d). A 4-inch 7440 glass wafer is bonded to the surface of silicon wafer with the rectangle array pattern for the formation of F–P cavities by a vacuum anodic bonding technique (Figure 4e). Vacuum anodic bonding, which directly affects the air tightness, pressure strength, and performance of pressure measurement, is vital for the sensor fabrication. A wet chemical etching method is used to reduce the thickness of silicon wafer (Figure 4f). Then, chemical mechanical polishing (CMP) is employed to further thin the silicon wafer for better surface roughness (Figure 4g). Repeating the process of spin-coating, lithography, developing, ICP etching, and photoresist removal, the thickness of the silicon upper F–P cavity is further reduced (Figure 4 h–j). Finally, the wafer is cut into small pieces via a scribing machine (Figure 4k) and the pressure-sensitive probe is fabricated (Figure 4l).

**Figure 5:**
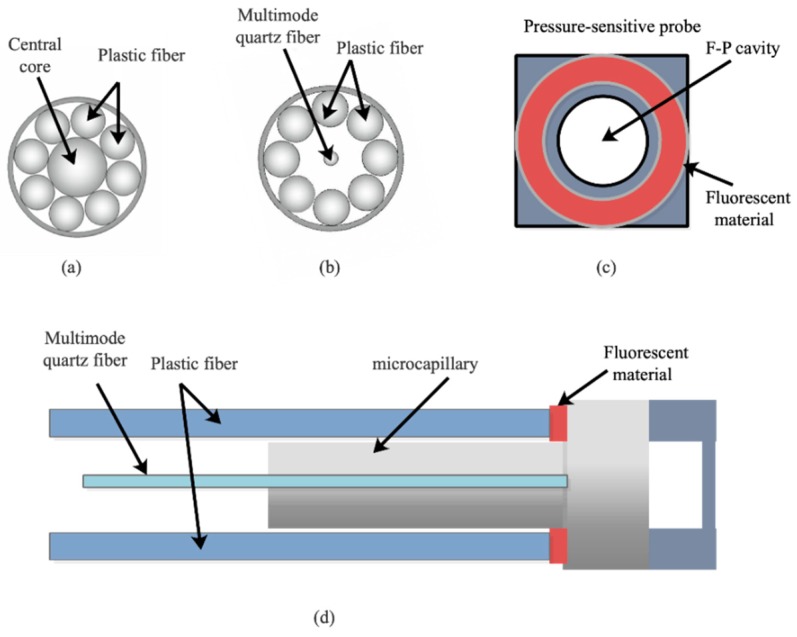
Manufacturing process of hybrid fiber-optic F–P sensor. (**a**) Multi-core plastic fiber, (**b**) replacing the central core, (**c**) mixture coating, (**d**) bonding.

After the pressure-sensitive probe is fabricated, it is bonded with an optical fiber, used for the light delivery, to form the hybrid fiber-optic F–P sensor, as depicted in Figure 5. The optical fiber (PRS3Y10, BOJKE, Shenzhen, China) is a multi-core plastic fiber (Figure 5a). The central core is peeled off from the fiber and a multimode quartz fiber, with a diameter of 62.5 μm, is inserted to the plastic fiber to serve as the central core (Figure 5b). The phosphor used as fluorescent material is mixed with UV glue. Next, the mixture is coated on the glass surface of the pressure-sensitive probe around the F–P cavity (Figure 5c). Finally, the optical fiber is aligned and bonded to the sensor with the aid of the microcapillary and mechanical clamp (Figure 5d), making the central core of optical fiber align with the F–P cavity center of the pressure-sensitive probe. 

## 3. Experimental Results and Discussions

**Figure 6:**
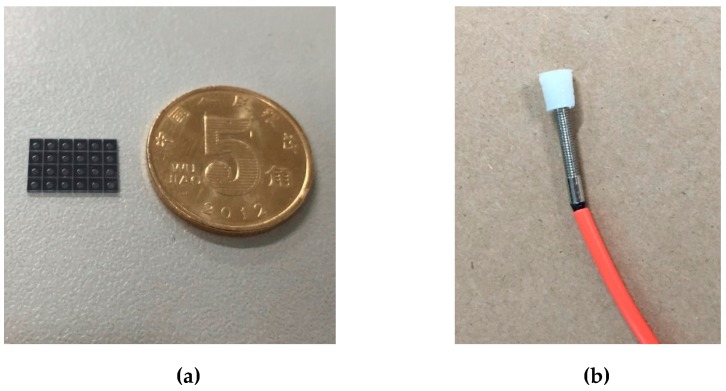
Images of (**a**) the pressure-sensitive probes and (**b**) the hybrid fiber-optic F–P sensor.

Figure 6a shows the pressure-sensitive probes before cutting. The radius and thickness of a single pressure-sensitive probe is 0.4 cm and 0.45 mm, respectively. The thickness of pressure-sensitive membrane is 26 μm and the length of the vacuum F–P cavity is 20 μm. The sensor probe and the optical fiber are clamped by a sensor holder to make the connection more reliable, as shown in Figure 6b. 

**Figure 7:**
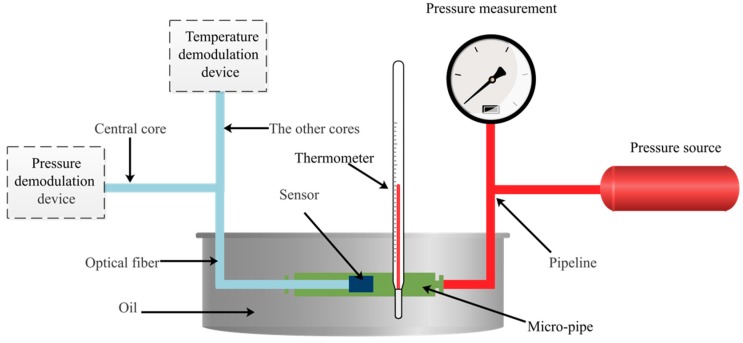
Experimental setup for testing sensor performance.

The experimental setup for testing the performance of the hybrid fiber-optic F–P sensor is depicted in Figure 7. A piston pressurization device (TS-5B/AK-2, Aerospace Aerodynamics Research Institute, Beijing, China) is used as the standard pressure source in the experimental setup. The pressure range of the pressure source is 0–500 KPa, with an accuracy rating of 0.5. The pressure and temperature demodulation devices are shown in Figure 2b and Figure 3, respectively. The micro pipe is heated by oil. A high-precision thermometer is utilized to measure the oil temperature.

**Figure 8:**
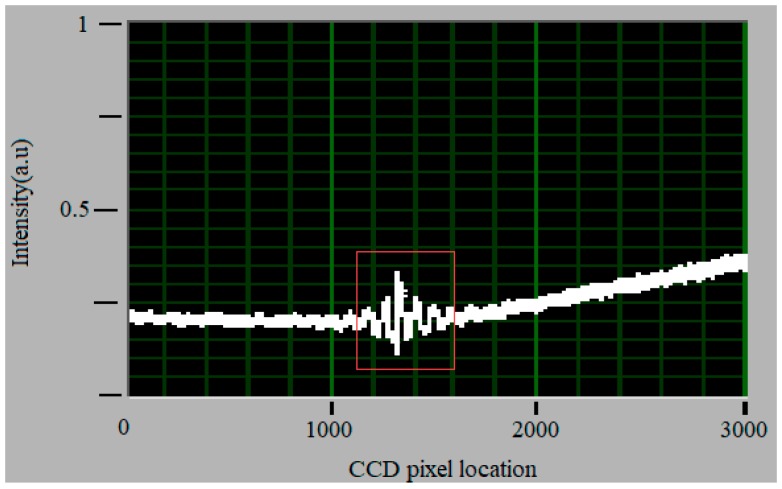
Interference patterns captured by the CCD of pressure demodulation device.

In the experimental setup, a hybrid fiber-optic F–P sensor was put into the micro pipe to test its performance. For the pressure measurement, the central core of optical fiber connected to the sensor is connected to the 1 × 2 coupler of pressure demodulation. The other cores of optical fiber are connected to the fiber coupler of fluorescence lifetime demodulation for temperature measurement. The interference patterns captured by the pressure demodulation device are shown in Figure 8. The horizontal axis shows the signal intensity. The vertical axis indicates the pixel location, which corresponds with the wedge thinness of the Fizeau interferometer. The pixel location of the interference signal can be obviously observed. 

**Figure 9:**
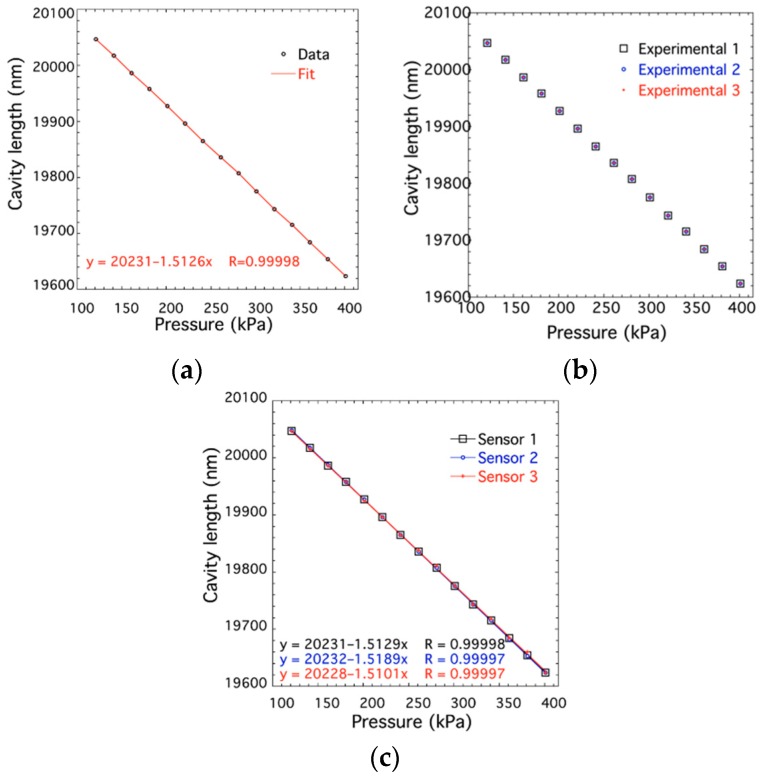
Calibration of the cavity length and the pressure applied on the sensor to test the (**a**) linearity, (**b**) repeatability, and (**c**) consistency.

The pressure applied to the sensor increased from 120 to 400 KPa at room temperature (25 °C). The cavity length of hybrid fiber-optic F–P sensor decreased when the pressure raised, the relationship of which is plotted in Figure 9a. It can be calculated that the sensitivity of cavity length was 1.5126 nm/KPa. The experiment was repeated for three cycles to verify the repeatability. The experimental results are shown in Figure 9b, which indicates that the pressure response to cavity length has a good repeatability. In order to test the consistency between various sensors the experiments were carried out on different sensors. The results are depicted in Figure 9c. The results of experiments mentioned above indicate that the hybrid fiber-optic F–P sensors have good linearity, repeatability, and consistency thanks to the MEMS technology.

For the fiber-optic F–P sensors, it is difficult enough to avoid the noise of the interference signal. Here, the wavelet algorithms are employed to denoise the signal for the improvement of stability. The wavelet transform can be defined as follows [33]:

(6) $$\left(W_{\varphi }\right)\left(a,b\right)={\left|a\right|}^{\frac{1}{2}}\int_{-\infty }^{+\infty }f\left(t\right)\bar{\psi }\left(\frac{t-b}{a}\right)dt$$

where *b* is the translation parameter, *a* is the scale expansion parameter, and $\psi_{\left(a,b\right)}\left(x\right)=\frac{1}{\sqrt{\left|a\right|}}\psi_{\left(a,b\right)}\left[\left(\frac{x-b}{a}\right)\right]$ is the continuous wavelet function, which depends on a pair of parameters (*a*, *b*) referred to as the wavelet.

The algorithm for eliminating the modulus maxima, controlled by noise and the noise threshold formula, when performing discrete binary wavelet transform on a signal can be expressed as follows [34]:

(7) $$T_{0}=\frac{log_{2}\left(1+2\sqrt{N}\right)}{J+Z}\times A$$

where *N* is the preset noise power, *J* is the largest scale obtained, Z is the unique constant, and *A* is the maximum extreme point amplitude. Using Equation (7), an appropriate threshold can be calculated to remove the modulus maxima, since the noise modes at these points can be considered to be dominant.

**Figure 10:**
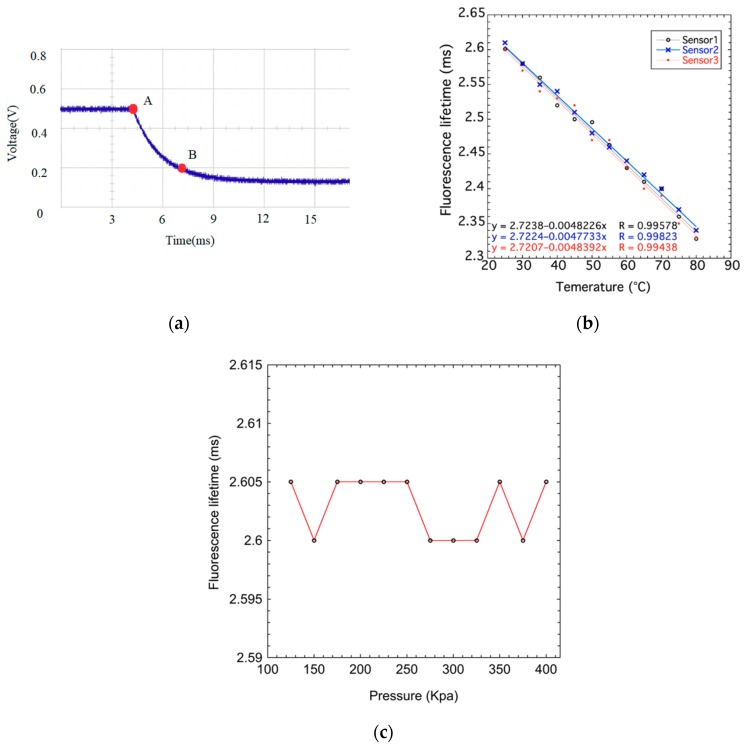
Characteristic of fluorescence lifetime in the hybrid fiber-optic F–P sensor. (**a**) Fluorescence decay curves at room temperatures, (**b**) Relationship between fluorescence lifetime and temperature, and (**c**) relationship between fluorescence lifetime and pressure.

The hybrid fiber-optic F–P sensor was heated by oil bath to test its temperature performance. The experiments were performed from 25 to 80 °C. The fluorescence lifetime from the sensor was recorded. Figure 10a shows the fluorescence decay curves at the temperatures of 25 °C, which is the original signal directly obtained from the temperature demodulation device detector. Point A is the peak of fluorescence intensity in one cycle and the fluorescence intensity of point B is 1/e of point A. Fluorescence lifetime is the period when the fluorescence intensity from point A falls to point B. The linear fitting of fluorescence lifetime and temperature was conducted for three different sensors. The result, shown in Figure 10b, indicates a good linear relation between the fluorescence lifetime and temperature and a good consistency between the different sensors. The sensitivity of fluorescence lifetime to temperature is about 0.0048 ms/°C.

Another group of experiments was performed to check the influence of pressure on the fluorescence lifetime. The fluorescence lifetime was measured under various pressures. The temperature was set to room temperature (25 °C) and kept constant during the experiment. The pressure–fluorescence lifetime curve is plotted in Figure 10c. It can be observed that the fluorescence lifetime is almost unchanged under different pressures. Accordingly, it can be concluded that the temperature measurement of hybrid fiber-optic F–P sensor cannot be affected by the pressure forced upon it. The fluorescence lifetime is only determined by the surrounding temperature.

**Figure 11:**
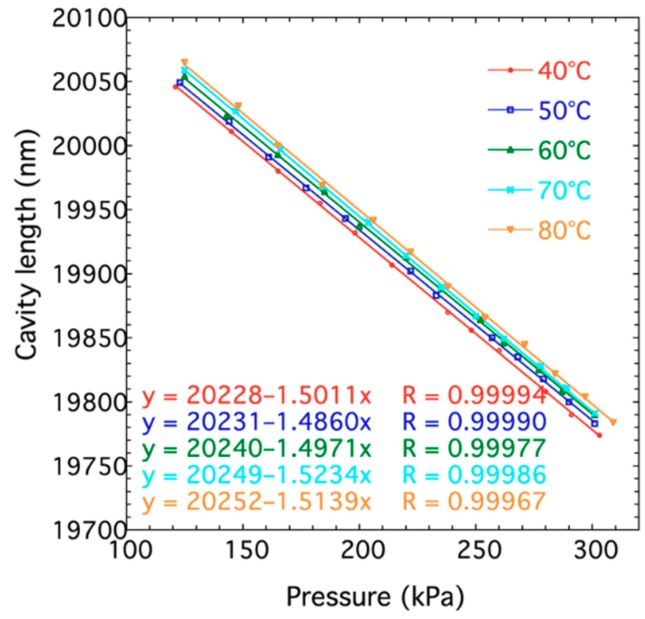
Relationship between cavity length and pressure at different temperatures.

Figure 11 shows the relationship between pressure and cavity length of the hybrid fiber-optic F–P sensor under different temperatures. The experimental results show that the cavity length has a good linear relationship with the pressure at constant temperature. However, the cavity length increases with the rise of surrounding temperature. The F–P cavity of the hybrid fiber-optic F–P sensor is constructed mainly by three kinds of materials, including polysilicon silicon, Pyrex 7740 glass, and UV glue. The polysilicon silicon and Pyrex 7740 glass are bonded by the UV glue. These three materials have different thermal expansion coefficients. Consequently, the thermal mismatch is caused when the temperature changes, which results in the variations of cavity length.

## 4. Conclusions

In conclusion, a hybrid fiber-optic F–P sensor for the simultaneous measurement of temperature and pressure is developed. Fluorescent material is introduced into the sensor to obtain temperature information in place of complex structures, making the sensor low-cost and easy to fabricate. An experimental setup that can measure the temperature and pressure simultaneously was set up to test the performance of sensors. It was demonstrated that the sensor achieved pressure measurement at the range of 120–400 KPa, at room temperature, with a sensitivity of 1.5113 nm/KPa. The linearity of pressure against cavity length variation was over 99.9%, even if the temperature changes. Moreover, a temperature measurement in the range of 25–80 °C was achieved. The sensitivity of fluorescence lifetime to temperature is 0.0048 ms/°C. We are certain that this sensor, which is low-cost, easily fabricated, compact sized, high-sensitivity, and good-linearity has many practical scientific and engineering applications in the field of pressure and temperature measurement.

## References

[1] Grattan K.T.V., Sun T. (2000). Fiber optic sensor technology: an overview. Sens. Actuators A Phys..

[2] Srivastava S.K., Verma R., Gupta B.D. (2011). Surface plasmon resonance based fiber optic sensor for the detection of low water content in ethanol. Sens. Actuators B Chem..

[3] Kersey A.D., Jackson D.A., Corke M. (1983). A simple fibre Fabry-Perot sensor. Opt. Commun..

[4] Rao Y.J. (2006). Recent progress in fiber-optic extrinsic Fabry–Perot interferometric sensors. Opt. Fiber Technol..

[5] Yoshino T., Kurosawa K., Itoh K., Ose T. (1982). Fiber-Optic Fabry-Perot Interferometer and its sensor applications. IEEE Trans. Microw. Theory Tech..

[6] Gangopadhyay T.K., Henderson P.J. (1999). Vibration: history and measurement with an extrinsic Fabry-Perot sensor with solid-state laser interferometry. Appl. Opt..

[7] Quirion M., Ballivy G. (2000). Laboratory investigation on Fabry-Perot sensor and conventional extens. Can. J. Civil. Eng..

[8] Márquez-Cruz V.A., Hernández-Cordero J.A. (2014). Fiber optic Fabry-Perot sensor for surface tension analysis. Opt. Express.

[9] Li C., Liu Q., Fan S., Peng X. (2015). Analyzing the temperature sensitivity of Fabry-Perot sensor using multilayer graphene diaphragm. Opt. Express.

[10] Liu H., Miller D.W., Talnagi J.W. (2003). Performance evaluation of Fabry-Perot temperature sensors in Nuclear Power Plant Measurements. Nucl. Technol..

[11] Tseng F.G., Lin C.J. (2003). Polymer MEMS-based Fabry-Perot shear stress sensor. IEEE Sens. J..

[12] Hill G.C., Melamud R., Declercq F.E., Davenport A.A., Chan I.H., Hartwell P.G., Pruitt B.L. (2007). SU-8 MEMS Fabry-Perot pressure sensor. Sens. Actuators A Phys..

[13] Masson J., St-Gelais R., Poulin A., Peter Y.A. (2010). Tunable fiber laser using a MEMS-Based in plane Fabry-Pérot filter. IEEE J. Quantum Electron..

[14] Li M., Wang M., Li H. (2006). Optical MEMS pressure sensor based on Fabry-Perot interferometry. Opt. Express.

[15] Kaur A., Xiao H., Huang J., Yuan L., Lan X., Zhang Y. (2013). High-temperature fiber-optic Fabry–Perot interferometric pressure sensor fabricated by femtosecond laser: erratum. Opt. Lett..

[16] Wu C., Fu H.Y., Qureshi K.K., Guan B.O., Tam H.Y. (2011). High-pressure and high-temperature characteristics of a Fabry-Perot interferometer based on photonic crystal fiber. Opt. Lett..

[17] Morris P., Hurrell A., Shaw A., Zhang E., Beard P. (2009). A Fabry-Perot fiber-optic ultrasonic hydrophone for the simultaneous measurement of temperature and acoustic pressure. J. Acoust. Soc. Am..

[18] Pang C., Bae H., Gupta A., Bryden K., Yu M. (2013). MEMS Fabry-Perot sensor interrogated by optical system-on-a-chip for simultaneous pressure and temperature sensing. Opt. Express.

[19] Bae H., Yu M. (2014). Miniature Fabry-Perot sensor with polymer dual optical cavities for simultaneous pressure and temperature measurements. Biochemistry.

[20] Pevec S., Donlagic D. (2012). Miniature all-fiber Fabry–Perot sensor for simultaneous measurement of pressure and temperature. Appl. Opt..

[21] Sun A., Qiao X.G., Jia Z.A., Li M., Zhao D.Z. (2005). Study of simultaneous measurement of temperature and pressure using double fiber Bragg gratings with polymer package. Opt. Eng..

[22] Kisała P., Cięszczyk S. (2015). Method of simultaneous measurement of two direction force and temperature using FBG sensor head. Appl. Opt..

[23] Wang W., Jiang X., Yu Q. (2012). Temperature self-compensation fiber-optic pressure sensor based on fiber Bragg grating and Fabry–Perot interference multiplexing. Opt. Commun..

[24] Wang D., Ding M., Pi H.Y., Li X., Yang F., Ye Q., Cai H.W., Wei F. (2018). Influence of intra-cavity loss on transmission characteristics of fiber Bragg grating Fabry–Perot cavity. Chin. Phys. B.

[25] Huang K., Li Q., Chen H. (2016). A switchable dual-wavelength fiber laser based on asymmetric fiber Bragg grating Fabry–Perot cavity with a SESAM. J. Mod. Opt..

[26] Zhao W., Wang J., Wang A., Claus R.O. (1998). Geometric analysis of optical fiber EFPI sensor performance. Smart Mater. Struct..

[27] Jerman J.H., Clift D.J., Mallinson S.R. A miniature Fabry-Perot interferometer with a corrugated silicon diaphragm support. Proceedings of the Solid-State Sensor and Actuator Workshop.

[28] Murphy K.A., Gunther M.F., Wang A., Claus R.O., Vengsarkar A.M. Extrinsic Fabry-Perot optical fiber sensor. Proceedings of the Optical Fiber Sensors Conference.

[29] Han M., Zhang Y., Shen F., Pickrell G.R., Wang A. (2004). Signal-processing algorithm for white-light optical fiber extrinsic Fabry-Perot interferometric sensors. Opt. Lett..

[30] Bhatia V., Murphy K.A., Claus R.O., Tran T.A., Greene J.A. (1999). Recent developments in optical-fiber-based extrinsic Fabry-Perot interferometric strain sensing technology. Smart Mater. Struct..

[31] Belleville C., Duplain G. (1993). White-light interferometric multimode fiber-optic strain sensor. Opt. Lett..

[32] Zhang Z., Grattan K.T.V., Palmer A.W. (1992). Fiber optic temperature sensor based on the cross referencing between blackbody radiation and fluorescence lifetime. Rev. Sci. Instrum..

[33] Jing M.A., Wen R.Q. (1999). Application of wavelet analysis to potical information processing. Acta Phys. Sin..

[34] Tan L., Ma J., Wang Q., Ran Q. (2001). Filtering theory and application of wavelet optics at the spatial. Appl. Opt..

